# Design Optimization of Piezocomposites Using a Homogenization Model: From Analytical Model to Experimentation

**DOI:** 10.3390/s24061957

**Published:** 2024-03-19

**Authors:** Corentin Camus, Pierre-Jean Cottinet, Claude Richard

**Affiliations:** Laboratoire LGEF—Laboratoire de Génie Électrique et Ferroélectricité, INSA Lyon, LGEF, UR682, 69621 Villeurbanne, France; pierre-jean.cottinet@insa-lyon.fr

**Keywords:** piezocomposite, smart-structure activation, piezoelectric transducers, analytical model

## Abstract

In the process of activating non-conductive smart-structures using piezoelectric patches, one possible method is to add a conductive layer to ensure electrical contact of both electrodes of the ceramic. Therefore, depending on the stiffness and the thickness of this layer, changes in the overall piezoelectric properties lead to a loss in the electromechanical coupling that can be implemented. The purpose of this work is to study the impact of this added electrode layer depending on its thickness. A model of the effect of this layer on the piezoelectrical coefficients has been derived from the previous approach of Hashimoto and Yamagushi and successfully compared to experimental data. This global model computes the variation of all the piezoelectric coefficients, and more precisely of $k_{31}$ or $d_{31}$ for various brass electrode volumes relative to the ceramic volume. A decrease in the lateral electromechanical coupling factor $k_{31}$ was observed and quantified. NAVY II PZT piezoelectric transducers were characterized using IEEE standard methods, with brass electrode thicknesses ranging from 50 to 400 microns. The model fits very well as shown by the results, leading to good expectations for the use of this design approach for actuators or sensors embedded in smart-structures.

## 1. Introduction

**Figure 1:**
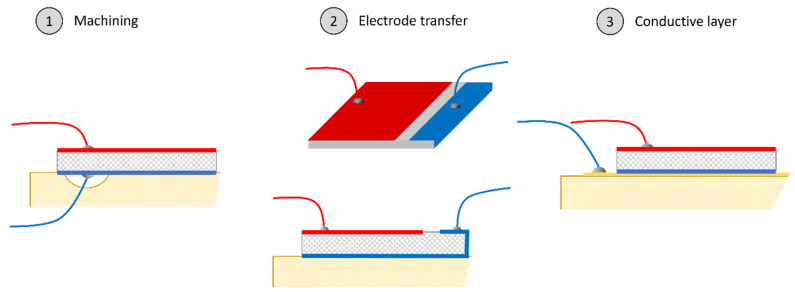
Three possible methods to ensure electrical contact for smart-structure activation using piezoceramic patches. Machining degrades the property of the structure, and electrode transfer reduces the poled volume, hence the piezo active portion. The third method is studied in this paper.

Piezoceramic patches are usually used as transducers on plane smart-structures because of their electromechanical conversion capabilities. Structure activation can be used for energy harvesting [1] or vibration damping [2]. These solutions require a ceramic poled along the thickness and bonded on the structure with wires connected with both positive and negative electrodes. With a metallic or conductive structure, the bonding can be made conductive by ensuring contact with the beam, which is generally the ground. In the case of a non-conductive host structure, multiple solutions can be proposed to enable this contact, such as machining the structure to enable a cable passage or transferring contact with an overlapping electrode on the top surface of the ceramic before poling, such as shown in Figure 1. The first solution affects the structural integrity while the second one induces a loss of the active part of the ceramic, as its polarization would not be effective among the entire volume. A third solution consists of adding a conductive layer between the structure to be activated and the transducer, making contact with the lower electrode available. Using this solution requires analysis of the effect of this conductive substrate on the global piezoceramic properties. In this paper, a homogenization model is proposed and fully described for the optimization of this conductive layer to optimize the transducer design. The results of the proposed model are exposed in Section 4, and are compared to various experimental configurations. The association of the conductive layer and the piezoceramic patch will be considered here as a sort of piezocomposite. 

Piezocomposites, associating a piezoelectric ceramic with another material to enhance their overall performance, have been well described by Newnham et al. [3], and since the 1980’s, models have been developed to predict their properties. As introduced by Newnham et al. [3], piezocomposites can be categorized according to their connectivity, which defines the way each phase ensures continuity along any of the three directions. Following this concept, a conductive layer associated with a ceramic plate can be mechanically and electrically considered as a 2–2 composite. Most of the proposed models rely on unidirectional simplifications and the implementation of Voigt or Reuss boundary conditions in order to derive a few significant homogenized properties. One of the first attempts to model the hydrostatic properties of piezo rubber from the NGK company was made by Hisao Banno in 1983 [4], using the modified cube theory relying on a cubic elementary representative volume. The same approach was followed by Newnham et al. [3] or Smith et al. [5,6] for hydrostatic or thickness mode 1.3 piezocomposite coupling predictions. These simple models were usually based on a few simple sets of equations. 

Few models considered the global relationships in the three directions to derive the whole homogenized properties, and few of them detailed the modeling method comprehensively allowing for an easy implementation. The reason is not really the difficulty but the complexity of the global analytical solution. More recently, one such global model relying on a numeric solution of the full set of equations was set up by Hashimoto and Yamagushi [7]. It was later used to focus on the global response of a 0–3 connectivity piezocomposite by Levassort et al. [8], and on the analysis of thermo-electromechanical properties [9].

In this paper, the global approach of Hashimoto and Yamaguchi [7] is developed and applied to derive the global properties of the effective material resulting from the association of the piezoelectric active layer and the passive electrode material resulting in a 2–2 connectivity composite.

It is shown that once the principal assumptions are synthetized as a set of equations in the three dimensions, the homogenization problem can be easily solved from the various materials properties with simple matrix calculations and inversions.

This article takes place with the context of aiming to optimize the activation of a cantilever beam using a plane piezoelectric transducer, and more specifically in deriving design rules for applying thick electrodes on a bulk ceramic. The analysis thus concentrates on the lateral coefficients and more specifically on the $k_{31}$, also known as the lateral electromechanical coupling coefficient related to the strains and stresses in the piezo element, along the beam direction, directly related to the bending of the host structure. Many works have been conducted on thick films on polyimide layers [10] or alumina [11], and on thin films with a silicon substrate [12]. They focused on modeling the clamping effect of the substrate on the apparent effective piezo element layer properties in comparison with bulk PZT. The property generally considered is the $d_{33}$ coefficient characterizing the longitudinal mode along the polarization direction of the piezoelectric material layer.

The evolution of the whole lateral mode-related properties is therefore comprehensively studied and exposed in this paper, where a two layer 2–2 piezocomposite (PZT + brass electrodes) configuration is analyzed both experimentally and theoretically. 

## 2. Model

### 2.1. Variable Definitions

The piezoelectric constitutive state equations describe the relation within a piezoelectric material of the mechanical strains and stresses and the electric fields and inductions. Equation (1) is one of the usual ways to write those relations, where $s^{E}$ is the stiffness matrix of the material under a constant electric field, $\varepsilon^{T}$ is the electrical permittivity matrix at constant stress, and d is the piezoelectric charge coefficient matrix. S and T are the strain and stresses vectors, whereas E and D are the electric field and induction vectors.

(1) $$\begin{array}{c} \left[\begin{array}{c} S\\ D \end{array}\right] \end{array}=\left[\begin{array}{cc} s^{E} & d^{t}\\ d & \varepsilon^{T} \end{array}\right]\left[\begin{array}{c} T\\ E \end{array}\right]$$

The matrix system (1) can be written as:

(2) $$\left[Y^{i}\right]=\left[A^{i}\right]\left[X^{i}\right]$$

The $s^{E}$ compliance matrix is a 6×6 matrix and the charge coefficient d is a 3×6 matrix, while the dielectric matrix has a 3×3 size. In these conditions, the assembled $A^{i}$ matrix should be 9×9. However, as shear is generally decoupled from longitudinal strain and stress coupling in the scope of linear behavior, shear will be here completely omitted, simplifying the problem and reducing the $A^{i}$ matrix size to 6×6. Note that shear modeling could be nevertheless considered, leading to a global modeling of the effective resulting properties. 

$\left[X^{i}\right]$ and $\left[Y^{i}\right]$ are defined as two 6 term row vectors as shown in the following:

(3) $$\left[X^{i}\right]=\left[\begin{array}{c} T_{1}^{i}\\ T_{2}^{i}\\ T_{3}^{i}\\ E_{1}^{i}\\ E_{2}^{i}\\ E_{3}^{i} \end{array}\right]\;\mathrm{and}\;\left[Y^{i}\right]=\left[\begin{array}{c} S_{1}^{i}\\ S_{2}^{i}\\ S_{3}^{i}\\ D_{1}^{i}\\ D_{2}^{i}\\ D_{3}^{i} \end{array}\right]$$

Note that the i superscript is a generic term and can be replaced by Z for the piezoelectric phase or P for the passive conductive layer. As this passive conductive layer has no piezoelectric coupling, the d coefficient will be a 3×6 null matrix and the conductive properties will be taken into account by considering an infinite permittivity. In the scope of this model, the permittivity will be set arbitrarily two orders above the magnitude of the piezo layer one. 

In the same way, the homogenized composite would be modelled as the following:

(4) $$\left[<Y>\right]=\left[<A>\right]\left[<X>\right]$$

where <A> is the homogenized properties matrix, and <X> and <Y> are the vectors assembling the globalized stress, electric field, strain, and induction, identical to those for both the piezoelectric phase or passive conductive layer.

This modeling approach relies on 24 variables constituting the 4 vectors defined in Equation (3) for both the piezoelectric phase and passive conductive phase. These variables will be assembled in a global variable vector $\left[B\right]$ defined in Equation (5). Note that the sub-vectors’ arrangement has been chosen under this form for a matter of symmetry.

(5) $$\left[B\right]=\left[\begin{array}{c} X^{P}\\ Y^{P}\\ Y^{Z}\\ X^{Z} \end{array}\right]$$

### 2.2. Composite Association Assumptions

**Figure 2:**
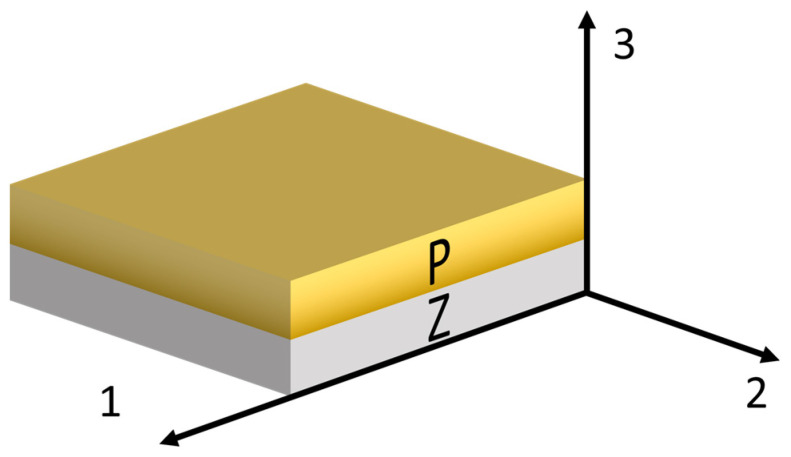
Elementary representative volume (ERV) of a 2–2 piezocomposite association of PZT and brass. P relates to the conductive brass layer and Z to the PZT material. This is represented in the reference coordinate system. As usual, the PZT layer is poled along direction 3.

Homogenization relies essentially on Voigt and Reuss assumptions. The bonding between the two layers is considered perfect. The elementary representative volume (ERV) is illustrated in Figure 2. Along directions 1 and 2, the strains and the electric fields of both phases are supposed to be identical and equal to the homogenized values. The homogenized composite stresses and electric displacements are the sum of each phase contributions relative to their respective volume fractions (parallel assumption). In the following, v is the volume ratio of the piezoelectric material. Along direction 3, these assumptions are inverted. Stress and electric displacement are considered identical, and the homogenized strain and electric field are a combination of each phase relative to their volume fraction (series assumption). These assumptions related to directions 1, 2, and 3 are then respectively defined by Equations (6)–(8), as shown in the following:

(6) $$\left\{\begin{array}{ccc} \langle T_{1}\rangle & = & vT_{1}^{Z}+\left(1-v\right)T_{1}^{P}\\ \langle E_{1}\rangle & = & E_{1}^{Z}\\ \langle E_{1}\rangle & = & E_{1}^{P}\\ \langle S_{1}\rangle & = & S_{1}^{Z}\\ \langle S_{1}\rangle & = & S_{1}^{P}\\ \langle D_{1}\rangle & = & vD_{1}^{Z}+\left(1-v\right)D_{1}^{P} \end{array}\right.$$

(7) $$\left\{\begin{array}{ccc} \langle T_{2}\rangle & = & vT_{2}^{Z}+\left(1-v\right)T_{2}^{P}\\ \langle E_{2}\rangle & = & E_{2}^{Z}\\ \langle E_{2}\rangle & = & E_{2}^{P}\\ \langle S_{2}\rangle & = & S_{2}^{Z}\\ \langle S_{2}\rangle & = & S_{2}^{P}\\ \langle D_{2}\rangle & = & vD_{2}^{Z}+\left(1-v\right)D_{2}^{P} \end{array}\right.$$

(8) $$\left\{\begin{array}{ccc} \langle T_{3}\rangle & = & T_{3}^{Z}\\ \langle T_{3}\rangle & = & T_{3}^{P}\\ \langle E_{3}\rangle & = & vE_{3}^{Z}+\left(1-v\right)E_{3}^{P}\\ \langle S_{3}\rangle & = & vS_{3}^{Z}+\left(1-v\right)S_{3}^{P}\\ \langle D_{3}\rangle & = & D_{3}^{Z}\\ \langle D_{3}\rangle & = & D_{3}^{P} \end{array}\right.$$

### 2.3. Model Matrix Assembly and Resolution

Phase material state Equation (2) and association assumptions Equations (6)–(8) are assembled in one global matrix $\left[K\right].$ The construction of this matrix will be described in four steps.

- The equality relations between physical quantities of the P and Z phases resulting from their continuity throughout the piezocomposite are assembled in Equation (9) into one 6×24 matrix $\left[M\right]$, defined as:

(9) $$\left\{\begin{array}{c} T_{3}^{Z}-T_{3}^{P}=0\\ E_{1}^{Z}-E_{1}^{P}=0\\ E_{2}^{Z}-E_{2}^{P}=0\\ S_{1}^{Z}-S_{2}^{P}=0\\ S_{2}^{Z}-S_{2}^{P}=0\\ D_{3}^{Z}-D_{3}^{P}=0 \end{array}\right.\Leftrightarrow \left[M\right]\left[B\right]=\left[0\right]$$

where

(10) $$[M]=\left[\begin{array}{cccccccccccccccccccccccc} 0 & 0 & 1 & 0 & 0 & 0 & 0 & 0 & 0 & 0 & 0 & 0 & 0 & 0 & 0 & 0 & 0 & 0 & 0 & 0 & -1 & 0 & 0 & 0\\ 0 & 0 & 0 & 1 & 0 & 0 & 0 & 0 & 0 & 0 & 0 & 0 & 0 & 0 & 0 & 0 & 0 & 0 & 0 & 0 & 0 & -1 & 0 & 0\\ 0 & 0 & 0 & 0 & 1 & 0 & 0 & 0 & 0 & 0 & 0 & 0 & 0 & 0 & 0 & 0 & 0 & 0 & 0 & 0 & 0 & 0 & -1 & 0\\ 0 & 0 & 0 & 0 & 0 & 0 & 1 & 0 & 0 & 0 & 0 & 0 & -1 & 0 & 0 & 0 & 0 & 0 & 0 & 0 & 0 & 0 & 0 & 0\\ 0 & 0 & 0 & 0 & 0 & 0 & 0 & 1 & 0 & 0 & 0 & 0 & 0 & -1 & 0 & 0 & 0 & 0 & 0 & 0 & 0 & 0 & 0 & 0\\ 0 & 0 & 0 & 0 & 0 & 0 & 0 & 0 & 0 & 0 & 0 & 1 & 0 & 0 & 0 & 0 & 0 & -1 & 0 & 0 & 0 & 0 & 0 & 0 \end{array}\right]$$

- The relation between the load on each piezocomposite materials and the global homogenized load $\langle X\rangle$ is summarized by matrix $\left[N\right]$:

(11) $$\left\{\begin{array}{cc} vT_{1}^{Z}+\left(1-v\right)T_{1}^{P} & =\langle T_{1}\rangle \\ vT_{2}^{Z}+\left(1-v\right)T_{2}^{P} & =\langle T_{2}\rangle \\ T_{3}^{Z} & =\langle T_{3}\rangle \\ E_{1}^{Z} & =\langle E_{1}\rangle \\ E_{2}^{Z} & =\langle E_{2}\rangle \\ vE_{3}^{Z}+\left(1-v\right)E_{3}^{P} & =\langle E_{3}\rangle \end{array}\right.\Leftrightarrow \left[N\right]\left[B\right]=\left[\langle X\rangle \right]$$

where $\left[N\right]$ is a 6×24 matrix defined as:

(12) $$\left[N\right]=\left[\begin{array}{cccccccccccccccccccccccc} 1-v & 0 & 0 & 0 & 0 & 0 & 0 & 0 & 0 & 0 & 0 & 0 & 0 & 0 & 0 & 0 & 0 & 0 & v & 0 & 0 & 0 & 0 & 0\\ 0 & 1-v & 0 & 0 & 0 & 0 & 0 & 0 & 0 & 0 & 0 & 0 & 0 & 0 & 0 & 0 & 0 & 0 & 0 & v & 0 & 0 & 0 & 0\\ 0 & 0 & 0 & 0 & 0 & 0 & 0 & 0 & 0 & 0 & 0 & 0 & 0 & 0 & 0 & 0 & 0 & 0 & 0 & 0 & 1 & 0 & 0 & 0\\ 0 & 0 & 0 & 0 & 0 & 0 & 0 & 0 & 0 & 0 & 0 & 0 & 0 & 0 & 0 & 0 & 0 & 0 & 0 & 0 & 0 & 1 & 0 & 0\\ 0 & 0 & 0 & 0 & 0 & 0 & 0 & 0 & 0 & 0 & 0 & 0 & 0 & 0 & 0 & 0 & 0 & 0 & 0 & 0 & 0 & 0 & 1 & 0\\ 0 & 0 & 0 & 0 & 0 & 1-v & 0 & 0 & 0 & 0 & 0 & 0 & 0 & 0 & 0 & 0 & 0 & 0 & 0 & 0 & 0 & 0 & 0 & v \end{array}\right]$$

- The matrix $\left[R\right]$ is composed similarly to the matrix $\left[N\right]$. It summarizes the homogenized composite strain and electric displacement as a function of each phase.

(13) $$\left\{\begin{array}{ccc} \langle S_{1}\rangle & = & S_{1}^{Z}\\ \langle S_{2}\rangle & = & S_{2}^{Z}\\ \langle S_{3}\rangle & = & vS_{3}^{Z}+\left(1-v\right)S_{3}^{P}\\ \langle D_{1}\rangle & = & vD_{1}^{Z}+\left(1-v\right)D_{1}^{P}\\ \langle D_{2}\rangle & = & vD_{2}^{Z}+\left(1-v\right)D_{2}^{P}\\ \langle D_{3}\rangle & = & D_{3}^{Z} \end{array}\right.\;\;\;\;\;\;\;\;\;\;\Leftrightarrow \;\;\;\;\;\;\;\;\;\;\;\left[\langle Y\rangle \right]=\left[R\right]\left[B\right]$$

where $\left[R\right]$ is a 6×24 matrix, defined as:

(14) $$\left[R\right]=\left[\begin{array}{cccccccccccccccccccccccc} 0 & 0 & 0 & 0 & 0 & 0 & 0 & 0 & 0 & 0 & 0 & 0 & 1 & 0 & 0 & 0 & 0 & 0 & 0 & 0 & 0 & 0 & 0 & 0\\ 0 & 0 & 0 & 0 & 0 & 0 & 0 & 0 & 0 & 0 & 0 & 0 & 0 & 1 & 0 & 0 & 0 & 0 & 0 & 0 & 0 & 0 & 0 & 0\\ 0 & 0 & 0 & 0 & 0 & 0 & 0 & 0 & 1-v & 0 & 0 & 0 & 0 & 0 & v & 0 & 0 & 0 & 0 & 0 & 0 & 0 & 0 & 0\\ 0 & 0 & 0 & 0 & 0 & 0 & 0 & 0 & 0 & 1-v & 0 & 0 & 0 & 0 & 0 & v & 0 & 0 & 0 & 0 & 0 & 0 & 0 & 0\\ 0 & 0 & 0 & 0 & 0 & 0 & 0 & 0 & 0 & 0 & 1-v & 0 & 0 & 0 & 0 & 0 & v & 0 & 0 & 0 & 0 & 0 & 0 & 0\\ 0 & 0 & 0 & 0 & 0 & 0 & 0 & 0 & 0 & 0 & 0 & 0 & 0 & 0 & 0 & 0 & 0 & 1 & 0 & 0 & 0 & 0 & 0 & 0 \end{array}\right]$$

- Material state Equation (2) can be written for both phase as follows:
  (15) $$\left[A^{i}\right]\left[X^{i}\right]-\left[Y^{i}\right]=0$$

That can finally be written as a function of *B*, as shown below:

(16) $$\left[\begin{array}{cccc} \left[A^{P}\right] & \left[-I\right] & \left[0\right] & \left[0\right] \end{array}\right]\left[B\right]=\left[0\right]$$

(17) $$\left[\begin{array}{cccc} \left[\begin{array}{c} 0 \end{array}\right] & \left[0\right] & \left[A^{Z}\right] & \left[-I\right] \end{array}\right]\left[B\right]=\left[0\right]$$

- Equations (9), (11), (16) and (17) can finally be assembled as shown below:

(18) $$\left[\begin{array}{c} \left[\begin{array}{cccc} \left[\begin{array}{c} A^{P} \end{array}\right] & \left[\begin{array}{c} -I \end{array}\right] & \left[0\right] & \left[0\right] \end{array}\right]\\ M\\ N\\ \left[\begin{array}{cccc} \left[\begin{array}{c} 0 \end{array}\right] & \left[0\right] & \left[A^{Z}\right] & \left[-I\right] \end{array}\right] \end{array}\right]\;\;\;\left[\begin{array}{c} X^{P}\\ Y^{P}\\ Y^{Z}\\ X^{Z} \end{array}\right]=\left[\begin{array}{c} \left[0\right]\\ \left[0\right]\\ \left[\langle X\rangle \right]\\ \left[0\right] \end{array}\right]\;\;\;\underset{\;}{\Leftrightarrow }\;\left[K\right]\left[B\right]=\left[\begin{array}{c} \left[0\right]\\ \left[0\right]\\ \left[\langle X\rangle \right]\\ \left[0\right] \end{array}\right]$$

with $\left[K\right]$, a 24×24 matrix summarizing this model’s equations and defined as the following:

(19) $$\left[K\right]=\left[\begin{array}{c} \left[\begin{array}{cccc} \left[A^{P}\right] & \left[-I\right] & \left[0\right] & \left[0\right] \end{array}\right]\\ \left[\;\begin{array}{ccccc} & & M & & \end{array}\;\right]\\ \left[\;\begin{array}{ccccc} & & N & & \end{array}\;\right]\\ \left[\begin{array}{cccc} \left[0\right] & \left[0\right] & \left[A^{Z}\right] & \left[-I\right] \end{array}\right] \end{array}\right]$$

This leads to

(20) $$\left[\langle Y\rangle \right]=\left[R\right]\cdot \left[B\right]=\left[R\right]{\left[K\right]}^{-1}\left[\begin{array}{c} \left[0\right]\\ \left[0\right]\\ \left[\langle X\rangle \right]\\ \left[0\right] \end{array}\right]=\left[U\right]\left[\begin{array}{c} \left[0\right]\\ \left[0\right]\\ \left[\langle X\rangle \right]\\ \left[0\right] \end{array}\right]$$

where

(21) $$\left[U\right]=\left[R\right]{\left[K\right]}^{-1}$$

Equation (21) contains the composite constitutive state equations defined by Equation (4). The homogenized properties can then be identified from the submatrix $\left[U_{truncated}\right]$ defined as

(22) $$\left[<Y>\right]=\left[U_{truncated}\right]\left[<X>\right]$$

where

(23) $$\left[U_{truncated}\right]=\left[U_{i,j}\right]=\left[\begin{array}{cc} \left[\langle s^{E}\rangle \right] & \left[\langle d^{t}\rangle \right]\\ \left[\langle d\rangle \right] & \left[\langle \varepsilon^{T}\rangle \right] \end{array}\right],\;i\in \left[1,6\right]\;and\;j\in \left[13,18\right]$$

$\left[U_{truncated}\right]$ is a 6×6 matrix containing the complete homogenized compliance dielectric and piezoelectric charge coefficient matrices, thus allowing for the computation of the piezoelectric coupling coefficients in the three directions. 

This approach will be implemented for a 2–2 configuration made of a NAVY II lead–zirconate–titanate (PZT) actuator plate and a brass electrode, and will be evaluated both numerically and experimentally in the following sections. 

**Figure 3:**
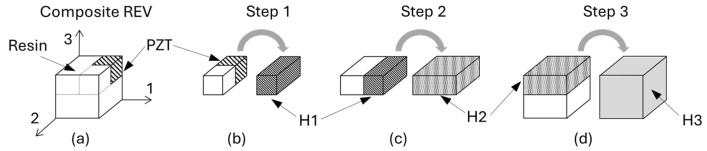
Homogenization in a multiple step process: (**a**) is the representative elementary volume of a 0.3 composite made with PZT particles in a resin; (**b**) is the first homogenization step associating the PZT and the resin to obtain material H1; (**c**) is the second homogenization associating material H1 and resin to obtain material H2; (**d**) is the last step associating the resin and material H2 to obtain the final homogenized material H3 representative of (**a**).

It is important to remark that this approach here implemented for a 2–2 composite, as depicted in Figure 2 and leading to the equation sets (6)–(8), can be derived for any orientation by adjusting the variable directions. Moreover 1–3, 3–3, or any composite configurations can be modeled in a few steps by just repeating this process according to the construction of a representative elementary volume. This is illustrated for a 0–3 composite in Figure 3. In this case, three steps are necessary.

### 2.4. Modeling and Experimental Data Processing

The model was implemented using MATLAB^®^ software, version 9.13.0 (R2022b) [13], using the physical characteristics of brass and PZT. Matrices $\left[A\right]$, $\left[M\right]$, $\left[N\right]$, and $\left[R\right]$ were created as defined in Section 2.2 and Section 2.3. The results of the modeling were derived from the numerical computation of $\left[K\right]$ and $\left[U\right]$. 

## 3. Sample Fabrication and Characterizations

### 3.1. Sample Preparation

The piezocomposite used in this study was made with P188, a NAVY II lead–zirconate–titanate (PZT) manufactured by Saint-Gobain [14] and characterized in laboratory conditions with a brass substrate. In order to get more comprehensive data, two batches of samples with various PZT volume fractions were made. The brass (CZ108/CW508L) was composed of 62% copper and 38% zinc, furnished by RS PRO, and followed the British standards on copper and copper alloys [15]. All physical properties are given in Table 1. All PZT plates were metallized using a silver paint cured at 600 °C for 4 h. They were consecutively polarized in an oil bath at room temperature under an electric field of 3 $\mathrm{kV}\cdot {\mathrm{mm}}^{-1}$ for two minutes.

### 3.2. Sample Batches Definition

**Figure 4:**
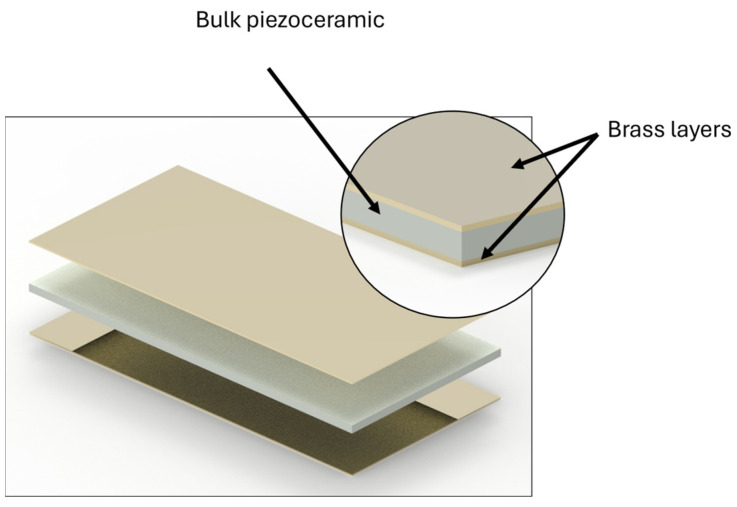
Schematics of the piezocomposite sandwich structure, where a PZT NAVY II piezoceramic is encapsulated between two layers of CZ108/CW508L brass. The layers are bonded using a hardener–resin epoxy mix (Araldite DBF and HY956). Epoxy resin is slowly cured at 60 °C.

**Figure 5:**
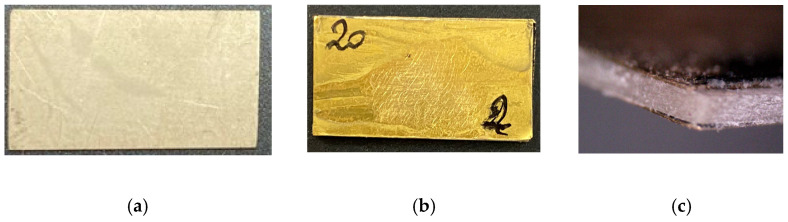
Photographs of the samples used: (**a**) is a bulk PZT plate with no brass electrode bonded; (**b**) is a piezocomposite made from a bulk PZT plate and a 200 μm brass layer on both sides, also shown in a lateral view in (**c**).

The impact of brass on the piezoelectric coefficients was studied by implementing various thicknesses. Complete sample configurations are given in Table 1 and physical properties for sample batch 1 and 2 are given in Table 2 and Table 3, respectively. Two brass sheets were bonded to the PZT layer using epoxy (84% Araldite DBF resin + 16% HY 956 hardener). This resulted in a sandwich, as shown in Figure 4. Epoxy resin was cured under a 0.2 MPa constant pressure at 60 °C for 4 h. As the curing temperature was limited, it is presumed that the stresses developing during the curing process were low enough to avoid any depolarization of the PZT. Note that two layers were used on both faces. According to the Voigt and Reuss assumptions, they will be modeled as a single brass layer with the corresponding volume fractions. Illustrations of samples with and without the brass layers are given in Figure 5. 

The choice of a non-conductive epoxy adhesive was motivated by its implementation simplicity. Apart from budget considerations, due to their high viscosity, conductive adhesives usually require a high pressure to obtain a low adhesive joint thickness, not always being compatible with the ceramic brittleness. Moreover, as developed by Lebrun [16], if the epoxy bonding layer is considered as a pure capacitance, a voltage drop of 20% would require an adhesive joint thickness lower than 0.5 µm for a 1 mm thick ceramic, thus inducing a large decrease in the capacitance. In practice, the faces of both PZT and brass can be considered as roughened surfaces, which enables both the adhesive resin to flow in the hollow parts of the surface and the electrical connection to be made through the peaks. Note that in this study, brass surfaces were previously scratched with sandpaper (P120, by Norton Abrasives—Saint-Gobain, Conflans-Sainte-Honorine, France) to induce a roughness, enabling both good bonding and electrical contact. The work of Kristiansen et al. [17] suggests that the presence of an electrical contact using non-conductive adhesive is highly dependent on the force applied during the process and the state of the surface. Imperfections on the surfaces induce irregularities in the thickness of the adhesive, ensuring electrical contact.

### 3.3. Measurements

As mentioned earlier, the piezoelectric plate associated with the brass electrodes is expected to work as a transducer in lateral mode in order to activate the bending of a cantilever beam or a plate structure. Measurements will therefore focus on the characterization of the properties directly related to the lateral mode: $k_{31},d_{31},s_{11}^{E},\varepsilon_{33}^{T}$. As the length and the width of the plates are different and much larger than the thickness, the measurement is made on the lowest resonance frequency corresponding to the material vibration mode along the larger dimension.

**Figure 6:**
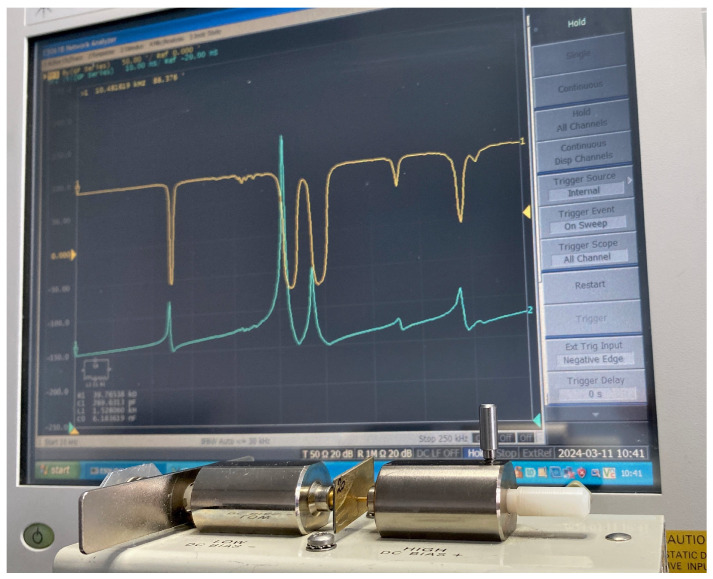
Photograph of the experimental set up used to characterize the piezoceramics and the piezocomposite. It is composed of an Agilent E5061B network analyzer and an Agilent 16034E test fixture.

Measurements are made following the IEEE standards [18]. Capacitance $C_{p}$ and dielectric loss $\tan\left(\delta \right)$ (@ 1kHz), as well as the impedance spectrum are measured using an Agilent E5061B impedance analyzer (Agilent, Santa Clara, CA, USA) with an Agilent 16034E test fixture (Agilent, Santa Clara, CA, USA). The experimental setup is illustrated in Figure 6. 

Resonant $\left(f_{s}\right)$ and antiresonant $\left(f_{p}\right)\;$frequencies of the first lateral mode are measured as the frequencies corresponding to the maximum of conductance $\left(G\right)$ and resistance $\left(R\right)\;$of the transducer, respectively. The density ρ is derived from the mass and sizes of each sample.

The lateral coupling coefficient $k_{31}$ is given by the following:

(24) $$k_{31}=\sqrt{\frac{\frac{\pi }{2}\frac{f_{p}}{f_{s}}}{\frac{\pi }{2}\frac{f_{p}}{f_{s}}-\tan\left(\frac{\pi }{2}\frac{f_{p}}{f_{s}}\right)}}$$

The vibration speed at constant electric field is expressed as a function of the length L of the sample by

(25) $$v_{b}^{E}=2\cdot L\cdot f_{s}$$

The elastic compliance $s_{11}^{E}$ is given by

(26) $$s_{11}^{E}=\frac{1}{\rho {\left(v_{b}^{E}\right)}^{2}}$$

The global permittivity at constant stress $\varepsilon_{33}^{T}\;$is derived from the capacitance and equal to the following:

(27) $$\varepsilon_{33}^{T}=\frac{C_{P}\cdot e}{S}$$

where e and S are the thickness and total surface of the sample, respectively. 

Finally, the lateral piezoelectric charge coefficient $d_{31}$ is obtained by

(28) $$d_{31}=k_{31}\sqrt{s_{11}^{E}\varepsilon_{33}^{T}}$$

## 4. Results and Discussion

### 4.1. Model Input Adjustment

Free PZT plates were first characterized according to this process, showing slight discrepancies with the nominal properties listed in Table 1. A comparison of these values is made in Table 4.

### 4.2. Results Analysis

Measurement and model results comparisons are given as a function of the additional brass electrode ratio σ, which represents the electrode portion added on a given PZT plate and is related to the volume fraction v as shown by the following:

(29) $$\sigma =\frac{1-v}{v}$$

**Figure 7:**
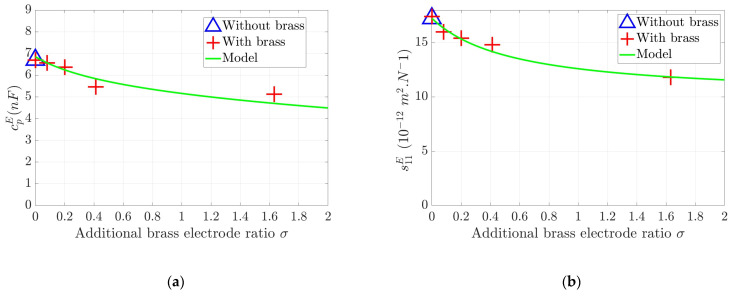
Comparison between measurements and modeled values for both variations in dielectric (**a**) and elastic properties (**b**) as a function of the additional brass electrode ratio σ. Triangle represents the initial value without brass.

Figure 7 illustrates the variation in the dielectric and elastic properties of the assembly as a function of the additional brass ratio. These properties are depicted by $C_{p}^{E}$ (Figure 7a) and $s_{11}^{E}$ (Figure 7b), for which the model is compared to the measurements. This figure illustrates and quantifies the clamp effect of the electrode on the ceramic, where it is shown that the thicker the brass layer, the lower the free capacitance and the dielectric permittivity. The same effect is causing the reduction in the global compliance due to the stiffness added by the brass electrode: because brass has a higher Young’s modulus than the PZT, this layer acts as a reinforcement resulting in a reduction in the global compliance of the assembly with the increase in the electrode layer thickness.

**Figure 8:**
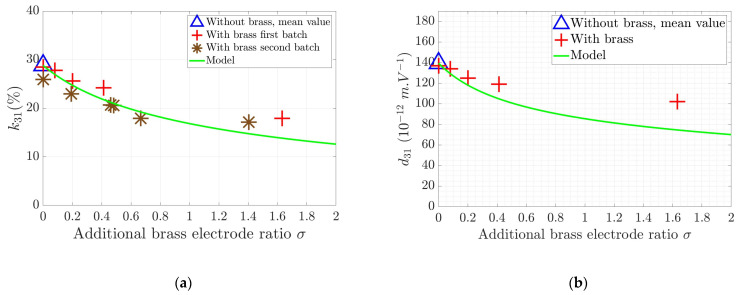
Lateral $k_{31}$ coupling coefficient (**a**) and $d_{31}$ charge coefficient (**b**) as a function of the additional brass electrode ratio σ and comparison with the model. Note that two batch samples were used to analyze the evolution of $k_{31}$.

Figure 8 illustrates the lateral piezoelectric coupling and charge coefficient variations due to the electrodes, which oppose the strain of the electroactive PZT layer. As stated before, the reinforcement due to the harder brass layer limits the electrically generated strain of the PZT ceramics, thus resulting in reduced piezoelectric coupling. Overall experimental and theoretical data appear to be in good agreement, showing the pertinence of the model to precisely quantify the electrode’s effect. Slight discrepancies appear for higher values of the additional brass ratio. This might be explained by the thickness of the bonding epoxy layer, considered as null in the model. As the epoxy resin is more compliant than the brass, some of the strain might be released in this layer resulting in a larger motion of the active PZT layer and an overall smaller impact of the brass-induced clamp on the piezocomposite. This effect is more marked as the added stiffness due to the electrode increases. Note that even with this aforementioned epoxy bond layer, electrical contact is ensured by the roughness of the brass electrode surface. 

**Figure 9:**
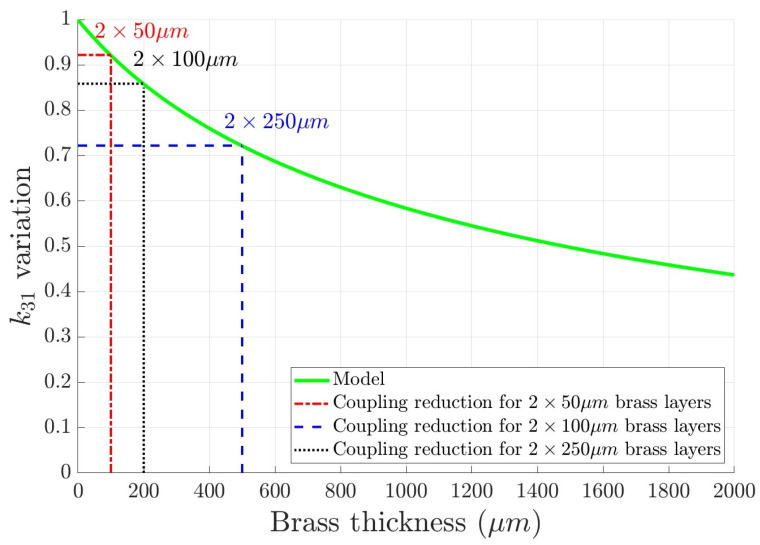
Variation of $k_{31}$ with the added brass thickness on a 1 mm thick PZT according to the proposed model. It is shown that two 50 μm thick brass layers bonded on each electrode of the ceramic reduces the coupling coefficient by less than 10%.

It is shown in Figure 9 that for a 1mm PZT actuator, two 50 μm thick brass electrodes induce a 10% decrease in the coupling coefficient, whereas a 500 μm electrode system reduces the coupling coefficient by less than 30%. These analyses lead to the conclusion that using a thin but still reasonable layer of brass of 50 to 100 μm, which is easy to handle and process, can be an efficient and functional solution to ensure an electrical contact between the lower electrode of the ceramic without altering its activation capability.

## 5. Conclusions

This paper has demonstrated and comprehensively described the application and the validity of a global numerical approach for the calculation of the full homogenized properties of a 2–2 brass-PZT piezocomposite, using a simple approach based on a series and parallel association configurations with basic matrix manipulations. The evolution of the lateral piezoelectric coefficients of a PZT patch versus the thickness of added brass electrodes was experimentally evaluated and compared to theoretical predictions. Two batches of ceramics were prepared and carefully characterized before and after bonding of brass. Experimental and theoretical results were shown to be in overall good agreement demonstrating both the reproducibility of the bonding process with epoxy resin and its feasibility with non-conductive resin. Moreover, it was shown that the practical bonding appears to be in accordance with the ideal series and parallel assumptions, as long as the added layer stiffness is not too high.

The given comprehensive description of this modeling method allows for an easy numerical implementation of the method for a large variety of configurations and materials, and is therefore useful to fully validate and justify guidelines or design rules governing the encapsulation of piezoelectric ceramic layers bonded on either conductive or isolating structures (glass, CFRP, or GFRP composites). 

## Figures and Tables

**Table 1:** Physical and piezoelectric properties for the NAVY II ceramic (manufactured by Saint-Gobain) and the brass layer. The brass dielectric coefficient is set to a value much higher than the PZT one in order to stand for the conductivity. Poisson’s ratio for the brass is an assumption, and Young’s modulus corresponds to a standard CZ108/CW508L brass alloy.

Ceramic (Navy II)		
$s_{11}^{E}$	15.44	$10^{-12}\;m^{2}\cdot N^{-1}$
$s_{12}^{E}$	−4.62	$10^{-12\;}m^{2}\cdot N^{-1}$
$s_{13}^{E}$	−7.84	$10^{-12}\;m^{2}\cdot N^{-1}$
$s_{33}^{E}$	20.09	$10^{-12}\;m^{2}\cdot N^{-1}$
$d_{13}^{E}$	−186	$10^{-12}\;m\cdot V^{-1}$
$d_{33}^{E}$	425	$10^{-12}\;m\cdot V^{-1}$
$\varepsilon_{11}^{T}/\varepsilon_{0}$	1970	
$\varepsilon_{33}^{T}/\varepsilon_{0}$	1850	
$T_{c}$	340	°C
**Brass (CZ108/CW508L)**		
$\varepsilon_{11}^{T}/\varepsilon_{0},\;\varepsilon_{22}^{T}/\varepsilon_{0},\varepsilon_{33}^{T}/\varepsilon_{0}$	$10^{4}$	
Young’s Modulus E	103.4	GPa
Poisson’s coefficient ν	$0.33\;\left(estimated\right)$	
Density ρ	8.4	$g\cdot {\mathrm{cm}}^{-3}$

**Table 2:** First sample batch: PZT layer is a 11 mm wide and 22 mm long rectangular plate with thicknesses ranging from 480 to 500 μm. The additional brass electrode ratio is defined later by Equation (29).

Brass Thickness (μm)	Ceramic Thickness (μm)	Additional Brass Electrode Ratio ($\frac{1-v}{v}$)
0	480	0%
2×20	500	8%
2×50	500	20%
2×100	485	41%
2×400	490	163%

**Table 3:** Second sample batch geometrical properties: The second batch of samples uses a 6 mm wide and 11 mm long rectangular PZT piezoceramics with thicknesses ranging from 500 to 650 μm. The additional brass electrode ratio is defined later in Equation (29).

Brass Thickness (μm)	Ceramic Thickness (μm)	Additional Brass Electrode Ratio ($\frac{1-v}{v}$)
0	520	0%
2×50	520	19.2%
2×80	500	32%
2×100	530	37.7%
2×130	540	48%
2×150	650	46.1%
2×200	600	66.7%
2×400	570	140.4%

**Table 4:** Measured values of the 11 and 22 compliances ($s_{11}^{E}$ and $s_{22}^{E}$) and the dielectric properties $d_{31}$, $d_{32}$, as well as the relative permittivity $\frac{\varepsilon_{33}^{T}}{\varepsilon_{0}}$ of the free bulk PZT compared to the nominal values proposed by St-Gobain. Slight discrepancies might be explained by a variation in the poling protocol.

Material Properties	Nominal Value (Saint-Gobain)	Measurement
$s_{11}^{E}=s_{22}^{E}\;\left(10^{-12}\;Pa^{-1}\right)$	15.44	17.24
$d_{31}=d_{32}\;\left(10^{-12}\;m\cdot V^{-1}\right)$	−186	−139
$\frac{\varepsilon_{33}^{T}}{\varepsilon_{0}}$	1850	1535

These measured values are used to adjust the input data of the model.

## Data Availability

Data are available on request due to privacy/ethical restrictions.
